# Osteosarcopenia: epidemiology, molecular mechanisms, and management

**DOI:** 10.3389/fendo.2025.1577758

**Published:** 2025-08-28

**Authors:** Yong-Tao Yi, Hao-Fan Zhao, Wei-Zhou Wang, Xi Li

**Affiliations:** Department of Orthopedics, The First Affiliated Hospital of Kunming Medical University, Kunming, Yunnan, China

**Keywords:** osteosarcopenia, osteoporosis, sarcopenia, chronic diseases, chronic inflammatory disease, mesenchymal stem cell therapy

## Abstract

Osteosarcopenia (OS), a recently recognized syndrome characterized by the simultaneous occurrence of osteopenia/osteoporosis and sarcopenia, has emerged as an important concept in clinical practice. This integrated framework provides a comprehensive view of the musculoskeletal system, addressing a previously underappreciated aspect of muscle health. OS notably increases the risk of falls, fractures, hospitalization, and mortality in elderly patients with chronic diseases. Despite its growing clinical relevance, OS remains underdiagnosed, and its classification as a distinct syndrome is not universally accepted. The persistently high global prevalence of chronic diseases, along with their substantial medical, economic, and social burdens, underscores the urgent need for updated prevention and management strategies. This review advocates for greater awareness and improved management of OS in patients with chronic diseases. It examines the relationship between OS and chronic conditions, emphasizing its epidemiology, adverse outcomes, diagnostic approaches, pathophysiology, and potential management strategies.

## 1 Background

Osteosarcopenia (OS) is defined as the concurrent presence of osteopenia/osteoporosis and sarcopenia (1), a concept first introduced by Duque et al. (1). It is a prevalent condition, particularly among the elderly. Prior to recognizing OS as a distinct syndrome, muscle-related disorders were often overlooked. However, acknowledging OS is crucial, as it significantly increases the risk of falls, fractures, hospitalization, and mortality in older individuals (2). Recent studies have also emphasized that OS contributes to the onset and progression of chronic diseases, thereby worsening outcomes for patients with these conditions (3, 4). Despite its growing significance, OS remains underrecognized in the management of chronic diseases, with much of the focus still placed solely on bone health. Given the global aging population, the prevalence of OS is expected to rise sharply in the coming years (5), underscoring the need for a more comprehensive approach to its role in chronic disease management.

Chronic diseases are defined as conditions lasting one year or more that require ongoing medical care, limit daily activities, or both (6). Often referred to as the “plague” of the 21st century, these conditions pose a significant threat to global health, second only to infectious diseases (7, 8) Cardiovascular diseases, chronic obstructive pulmonary disease (COPD), diabetes, chronic kidney disease, and chronic liver disease have long been major burdens on healthcare systems, collectively contributing to more deaths worldwide than all other causes combined (7). Furthermore, the limited treatment options available have resulted in recurrent hospitalizations, driving up medical costs and creating substantial challenges for healthcare economies, particularly in certain Asian countries (9). In response to these pressing issues, there is an urgent need to update and improve prevention and management strategies for chronic diseases.

The management of chronic diseases is centered on preserving physiological reserves and maintaining long-term homeostasis. The musculoskeletal (MSK) system not only facilitates locomotion but also plays a critical role in functional metabolism and nutrient storage (10). The onset of OS significantly undermines the health and stability of patients with chronic diseases. As such, there is an urgent need to reassess and expand our understanding of OS to develop more effective and up-to-date management strategies. This review aims to explore the impact of OS on chronic diseases, focusing particularly on its diagnosis, pathophysiology, and potential management approaches.

## 2 Osteosarcopenia: epidemiology and clinical diagnosis

### 2.1 Clinical diagnosis

Currently, there are no universally accepted diagnostic criteria for OS. Its clinical diagnosis is typically based on the concurrent presence of osteopenia/osteoporosis and sarcopenia, as identified through ongoing research and expert consensus. In clinical practice, OS is diagnosed when both of these conditions are present simultaneously.

#### 2.1.1 Diagnosis of osteopenia/osteoporosis

The World Health Organization (WHO) defines osteoporosis using bone mineral density (BMD) measurements obtained through Dual-Energy X-ray Absorptiometry (DXA). Osteoporosis is diagnosed when BMD is 2.5 standard deviations (SD) or more below the young adult reference mean (11). The T-score, which reflects BMD values at key sites such as the lumbar spine and femoral neck, is used to assess both osteopenia and osteoporosis (12). A T-score of ≥ -1.0 SD is considered normal; a T-score between -2.5 SD and -1.0 SD indicates osteopenia; and a T-score of ≤ -2.5 SD signifies osteoporosis (13). This classification is widely accepted and utilized globally.

#### 2.1.2 Diagnosis of sarcopenia

In 2018, the European Working Group on Sarcopenia in Older People (EWGSOP) established diagnostic criteria for sarcopenia. The key indicators for muscle strength include: grip strength <27 kg for men and <16 kg for women, or a chair stand test time >15 seconds for more than five repetitions. For muscle mass, the threshold is defined as appendicular skeletal muscle mass <20 kg for men and <15 kg for women. Regarding muscle function, a gait speed ≤0.8 m/s is considered critical. The most reliable methods for assessing muscle mass in clinical settings are bioelectrical impedance analysis (BIA) and Dual-Energy X-ray Absorptiometry (DXA), with DXA regarded as the gold standard for diagnosis (14). The recommended cutoff values for muscle mass measurement are 7.0 kg/m² for men and 5.4 kg/m² for women using DXA, and 7.0 kg/m² for men and 5.7 kg/m² for women using BIA (15).

### 2.2 Epidemiology

The prevalence of OS increases with age among community-dwelling individuals. In men, it ranges from 14.3% in those aged 60–64 to 59.4% in those aged ≥75 years; in women, it ranges from 20.3% in those aged 60–64 to 48.3% in those aged ≥75 years. Overall, the prevalence tends to be higher in women than in men (5). These findings align with the aging process, suggesting that as the global population ages, the prevalence of OS is expected to rise, although it may vary across regions or ethnic groups.

## 3 The effect of osteosarcopenia on chronic diseases

### 3.1 Chronic liver disease

OS significantly increases the risk and worsens the prognostic outcomes of chronic liver disease. Recent studies have shown that OS is associated with higher mortality rates and a reduced quality of life (QOL) in patients with cirrhosis (LC) (16). Additionally, OS exacerbates severe late-stage liver disease complications, including hepatic encephalopathy and infections (17). A retrospective study by Chisato Saeki et al. found that OS significantly elevated the risk of mortality in cirrhosis patients compared to controls (hazard ratio (HR): 4.798; 95% Confidence Intervals (CI): 1.885–12.212; p = 0.001), making it a critical independent prognostic factor for these individuals (18). Furthermore, Saeki’s study highlighted that in patients with primary biliary cholangitis and OS, the incidence of vertebral fractures was as high as 55.6%, which was significantly greater than in other groups (19). Additionally, a prospective cohort study revealed that the prevalence of OS increases with the progression of liver fibrosis. Notably, 7.0% of chronic hepatitis C patients without cirrhosis developed OS before the onset of cirrhosis (20). This suggests a potential association between OS and the progression of liver fibrosis, although the underlying pathophysiological mechanisms warrant further exploration.

### 3.2 Cardiovascular diseases

OS and cardiovascular diseases are both age-related conditions that share numerous common risk factors (21). A study involving 70,697 participants found that OS was independently associated with a 17% increased risk of heart failure (HF) events (22). Additionally, OS was strongly and independently linked to major electrocardiographic abnormalities in the elderly and significantly correlated with the onset and progression of coronary heart disease (23). Previous studies have also demonstrated that OS increases the risk of falls and fractures (24). Clinically, elderly individuals with chronic heart disease are at heightened risk of falls and fractures. Among adults with chronic heart conditions like heart failure (HF), the fall rate is 43%, compared to approximately 30% in those with other chronic diseases (25). This increased risk cannot be solely attributed to low cardiac output and polypharmacy. The significant role of OS as a common condition in elderly individuals with chronic heart disease may often be underrecognized. However, research exploring the relationship between these two conditions remains limited, and the mechanisms through which OS contributes to the heightened cardiovascular risk warrant further investigation.

### 3.3 Chronic kidney disease

OS is strongly associated with poor health outcomes and declining renal function in patients with chronic kidney disease (CKD) (26). A study by Yuta Nakano et al. demonstrated that patients with OS experienced significantly worse primary adverse outcomes compared to those with a single condition (HR: 3.28; 95% CI: 1.52-7.08) and had an elevated risk of renal composite adverse outcomes (HR: 2.07; 95% CI: 1.10-3.89) (26). Another study reported that in CKD patients, those with OS had a 33% higher risk of mortality compared to those without OS (HR: 1.33; 95% CI: 1.07–1.66; P = 0.011), and the likelihood of progression to end-stage renal disease (ESRD) was more than twice as high as in the control group (HR: 2.08; 95% CI: 1.53–2.82; P < 0.001) (27). Furthermore, patients receiving renal replacement therapy are often in a more weakened and energy-deprived state (28). Since MSK provides essential energy and functional support for these patients, OS poses a potentially “fatal” threat to individuals with chronic kidney disease.

### 3.4 Chronic respiratory diseases

OS significantly increases the risk of hospitalization and mortality in patients with chronic obstructive pulmonary disease (COPD) (29). COPD patients often experience long-term overinflation and heightened dyspnea due to worsening airflow obstruction. The weakening of the musculoskeletal system (MSK) in these patients can lead to a reduction in type I muscle fibers, decreased oxidative enzyme activity, and reduced muscle capillaries (30). all of which exacerbate breathing difficulties. COPD is particularly prevalent among the elderly, and in its advanced stages, systemic cachexia is frequently observed. This condition is marked by mitochondrial damage and oxidative stress resulting from prolonged hypoxia, contributing to a negative nitrogen balance and systemic depletion. OS-induced frailty, combined with the decline in respiratory muscle function, may worsen the severity of dyspnea in COPD patients, leading to more frequent hospitalizations.

**Figure 1:**
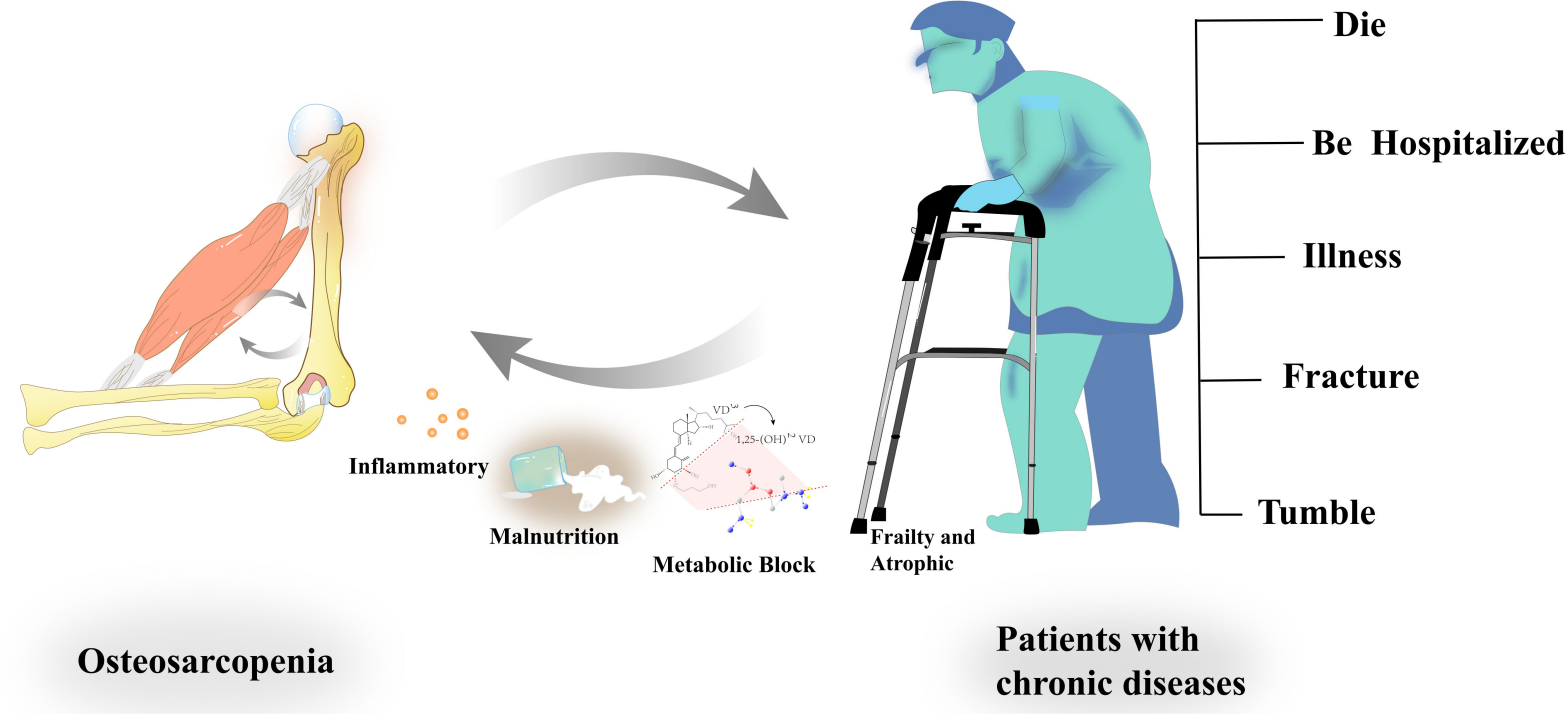
Interaction between osteosarcopenia and chronic diseases. Sarcopenia is a significant risk factor for increased prevalence, hospitalization rates, mortality, fractures, and falls, particularly in elderly populations. Chronic diseases serve as key modulators in the intricate interplay between osteoporosis and sarcopenia. These conditions accelerate the progression of Osteosarcopenia by promoting systemic inflammation, hormonal imbalances, and metabolic dysfunction, which in turn exacerbate the detrimental effects of Osteosarcopenia on overall health. The mutual interaction between Osteosarcopenia and chronic diseases worsens both skeletal and muscular integrity, creating a cycle of deterioration that significantly impacts patient prognosis.

In summary, OS plays a crucial role in mediating the occurrence of various adverse outcomes in chronic diseases, underlining its critical importance in the long-term management of these conditions. Despite its significance, OS remains insufficiently recognized and addressed in the management of chronic diseases, particularly in conditions such as diabetes and cancer. It is strongly recommended that OS be actively identified (**Table 1**). Furthermore, prior studies suggest that chronic diseases may contribute to the development of OS through mechanisms such as systemic inflammation, metabolic disorders, long-term polypharmacy, and frailty. Recent research highlights the potential interactions between these factors (**Figure 1**). However, research on how OS influences the onset of chronic diseases is still in its early stages and remains a challenging area of investigation.

**Table 1:** Research on the impact of osteosarcopenia on chronic diseases.

Author/year	Article type	Chronic disease	Results	Cite
Chisato Saeki et al.; 2023.	Retrospective Study	Liver Cirrhosis	Osteosarcopenia is significantly associated with mortality in patients with liver cirrhosis.	(18)
Tatiana Bering et al.;2018	Retrospective Study	Chronic Hepatitis C	The prevalence of osteosarcopenia increases concurrently with the progression of liver fibrosis.	(20)
Chisato Saeki et al.;2021	Retrospective Study	Primary Biliary Cholangitis	The prevalence of vertebral fractures in patients with primary biliary cholangitis and osteosarcopenia is as high as 55.6%, significantly higher compared to other groups.	(19)
Zhenjie Gu et al.;2020	Meta-Analysis	Heart Failure	Osteoporosis is significantly associated with an increased risk of heart failure.	(22)
Ramin Heshmat et al.;2021	Cross-Sectional Study	Cardiovascular Disease	Sarcopenia and its components are associated with electrocardiographic abnormalities in elderly Iranians. Sarcopenia may be identified as a specific risk factor for future cardiovascular events.	(23)
Yuta Nakano et al.;2024	Prospective Cohort Study	Chronic Kidney Disease	Osteosarcopenia is associated with poor survival rates and declined renal function in elderly CKD patients.	(26)
Thomas J Wilkinson et al.;2021	Cross-Sectional Study	Chronic Kidney Disease	The presence of sarcopenia increases the risk of death and end-stage renal disease (ESRD) in patients with chronic kidney disease.	(27)
Kai Ma et al.;2022	Review.	chronic obstructive pulmonary disease	ostosarcopenia is widely observed as a common feature of several chronic diseases, including aging and chronic obstructive pulmonary disease (COPD).	(30)

## 4 The pathogenesis of osteosarcopenia associated with chronic disease

### 4.1 NF-κB signaling pathway

The NF-κB signaling pathway plays a pivotal role in the development and metabolic processes of both bone and muscle cells, making it a key pathway in the pathogenesis of OS. Chronic systemic inflammation (SCI) is a central factor in the progression of this condition (31). As a well-established inflammatory signaling pathway, the activation of NF-κB leads to the production of pro-inflammatory cytokines that promote muscle degradation and bone resorption. Consequently, the progression of OS is not only driven by the NF-κB pathway itself, but is also significantly aggravated by chronic inflammatory diseases.

#### 4.1.1 RANK/RANKL-OPG—NF-κB

The receptor activator of NF-κB (RANK)-RANK ligand (RANKL)-osteoprotegerin (OPG) system is central to the regulation of bone remodeling (32). RANK, RANKL, and OPG are all components of the tumor necrosis factor (TNF) receptor superfamily. RANKL, a soluble ligand primarily produced by osteoblasts and T cells, inhibits osteogenesis while promoting osteoclast differentiation. In this system, RANKL binds to its receptor RANK, triggering downstream signaling molecules, such as colony-stimulating factor 1 receptor (CSF-1) and NF-κB receptors, which initiate osteoclast differentiation (32). OPG, another receptor for RANKL, inhibits osteoclastogenesis and bone loss by binding to RANKL, thus blocking the RANK-RANKL signaling cascade (33).

Additionally, RANKL is expressed in skeletal muscles, where its activation suppresses myogenic differentiation and induces muscle atrophy through the regulation of the NF-κB signaling pathway (34). Hamoudi et al. found that anti-RANKL treatment could shift macrophages to the M2 phenotype and inhibit NF-κB activation in Duchenne muscular dystrophy, thereby protecting muscles from chronic inflammation and improving their mechanical properties (35). They also observed that OPG knockout mice exhibited selective atrophy of fast-twitch type IIb muscle fibers, and C2C12 myotubes exposed to RANKL showed a reduction in cross-sectional area (34). These findings suggest that the OPG/RANKL/RANK-NF-κB axis plays a pivotal role in the crosstalk between bone and muscle, further linking bone loss and muscle atrophy in OS (36).

Systemic chronic inflammation leads to elevated TNFα levels, which directly promote osteoclast formation in the early stages by stimulating the expression of the colony-stimulating factor-1 receptor gene. This process increases osteoclast precursors and enhances osteoclastogenesis (37) (38) (39). Additionally, IL-6 and IL-1 can directly enhance osteoclast activity or indirectly increase RANKL production in osteocytes, further facilitating osteoclast activation and supporting bone resorption mediated by osteoblasts (40) (41) (42) (4).Recent studies have identified a novel mechanism linked to mutations in the GPNMB gene in COPD, which is associated with osteoporosis. The GPNMB gene encodes a type I transmembrane protein that acts as a positive regulator of osteoblast function. Research has shown that mutations in the GPNMB gene disrupt bone homeostasis by mediating abnormal RANKL levels. Furthermore, these mutations exacerbate pulmonary inflammation in COPD patients by modulating MYC expression, adding another layer of complexity to the relationship between bone metabolism, chronic inflammation, and pulmonary disease (43). In summary, the RANK/RANKL-OPG-NF-κB pathway plays a critical role in both bone loss and muscle atrophy. Osteoblasts, as a major source of RANKL, are central to this pathway, underscoring their significant role in the complex “bone-muscle crosstalk” that contributes to the development of OS. This highlights the potential of osteoblast function as an important area of research, especially in the context of chronic diseases.

#### 4.1.2 NF-κB—UPS

The ubiquitin-proteasome system (UPS) plays a central role in regulating protein degradation, selectively eliminating damaged or misfolded proteins, and maintaining cellular homeostasis (44). In bone remodeling, the UPS significantly impacts osteoblast function, with several E3 ubiquitin ligases involved in negatively regulating bone metabolism (45). For example, WWP1, a key negative regulator of osteoblast activity, promotes the degradation of the osteogenic gene Runx2, inhibiting the differentiation of osteoprogenitors into mature osteoblasts (46). This process is tightly regulated by the TNF-α-mediated NF-κB signaling pathway (46). However, NF-κB may not be the only pathway contributing to the suppression of osteogenic potential in bone marrow stromal cells (BMSCs).

At the same time, the UPS is also the primary for myofibril degradation. The UPS operates by attaching ubiquitin to substrate proteins, marking them for degradation by the 26S proteasome. E3 ubiquitin ligases, such as Atrogin-1 and MuRF-1, serve as rate-limiting factors in this pathway, and their upregulation is associated with muscle wasting in various chronic conditions (47). Chronic inflammatory factors, such as TNF-α, contribute to muscle atrophy by activating the NF-κB signaling pathway, which leads to the translocation of NF-κB from the cytoplasm to the nucleus (30) (48).This activation enhances the expression of E3 ligases like Atrogin-1 and MuRF-1, thereby increasing the degradation of myofibrillar proteins in skeletal muscle.

In summary, inflammatory factors such as TNF-α, IL-6, and IL-1 play a crucial role in the NF-κB-mediated development of OS (49). Since NF-κB is central to linking chronic diseases with OS, targeting this pathway could provide promising strategies for rethinking and treating the condition.

### 4.2 GH-IGF-1 axis

Insulin-like growth factor 1 (IGF-1) is synthesized by nearly all tissues and plays a pivotal role in mediating cell growth, differentiation, and transformation (50). Its secretion is primarily regulated by the growth hormone (GH) axis, with the liver serving as the primary source of circulating IGF-1 production and secretion. IGF-1 primarily by enhancing protein synthesis via the PI3K/Akt/mTOR signaling pathway (51). While current studies indicate that IGF-1 exhibits genetic polymorphisms, both systemic and locally produced skeletal IGF-1 are crucial for regulating bone mass formation and maintenance by promoting bone matrix synthesis and inhibiting bone resorption (52–54). Moreover, IGF-1 is increasingly recognized as an anabolic hormone that governs skeletal modeling and remodeling throughout the lifespan.

IGF-1 is involved in several anabolic pathways in skeletal muscle (55). In an animal study, localized expression of the IGF-1Ea or IGF-1Eb isoforms within muscle tissue was shown to counteract sarcopenia without causing significant side effects in other tissues or organs, nor did it affect the animals’ lifespan (56). This suggests that local expression of IGF-1 may play a role in preserving the youthful state of muscle tissue. However, it is important to recognize that OS frequently coexists with aging. The decline in IGF-1 levels associated with both aging and chronic diseases is a key factor contributing to the pathogenesis of OS.

In aging or end-stage liver disease, hepatic dysfunction, along with a reduction in growth hormone (GH) receptors, leads to decreased serum levels of insulin-like growth factor 1 (IGF-1) (57). Concurrently, impaired synthesis of branched-chain amino acids (BCAAs) disrupts BCAA-activated mTOR signaling, consequently interfering with glycogen and protein synthesis (58). mTOR, an atypical protein kinase, plays a central role in regulating growth and metabolism in response to nutrients, growth factors (such as IGF), and cellular energy status (59). Decreased BCAA levels further exacerbate the suppression of IGF-1 secretion. Moreover, prior studies have demonstrated that compensatory increases in angiotensin II in patients with chronic heart failure (CHF) contribute to sarcopenia and suppress osteoblast function by disrupting IGF-1 signaling (60). Zhang et al. identified a novel mechanism in a CKD mouse model, in which impaired IGF-1 receptor signaling compromised satellite cell function during muscle regeneration, leading to muscle fibrosis (61). Furthermore, glucocorticoids (GCs), frequently used in the long-term management of various chronic conditions, influence the regulation of the GH-IGF-1 axis. On one hand, GCs induce osteoblast apoptosis by inhibiting Wnt signaling and negatively regulating IGF-1 signaling, thereby hindering osteoblast precursor differentiation and maturation (62). On the other hand, GCs enhance osteoclast activity by modulating the RANKL/RANK/OPG pathway, which promotes bone resorption (63). With regard to muscle tissue, GCs not only inhibit IGF-1 production but also increase myostatin expression, thereby attenuating muscle protein synthesis (30). In conclusion, the GH-IGF-1 axis plays a predominantly anabolic role in the regulation of both bone and muscle health. However, factors that impair IGF-1 synthesis are significant contributors to the pathogenesis of OS. Consequently, IGF-1 replacement therapy may offer a promising therapeutic strategy for managing chronic diseases associated with OS.

### 4.3 Vitamin D

Vitamin D is widely recognized as a critical regulator of bone formation and has long been a central focus in the prevention and treatment of osteoporosis. Factors such as poor nutrition and insufficient sunlight exposure are commonly associated with impaired vitamin D synthesis and metabolism, often leading to vitamin D deficiency (64). This deficiency directly affects bone mineralization and matrix formation, resulting in abnormal bone growth and development. A recent study by Michael Booth and colleagues identified a high prevalence of vitamin D deficiency among young orthopedic trauma patients, suggesting an inverse relationship between vitamin D levels above 30 ng/mL and BMD (65).

Furthermore, recent studies have confirmed that decreased vitamin D levels are associated with an increased risk of sarcopenia. A study by Mori K et al. demonstrated that calcitriol could alleviate skeletal muscle changes induced by CKD (66, 67) (68). First, Vitamin D plays a key role in muscle health by promoting myoblast self-renewal and maintaining the satellite stem cell pool, through the regulation of the Forkhead box O (FOXO)3 and Notch signaling pathways (69). Second, the active form of vitamin D, 1,25(OH)2D, binds to the vitamin D receptor (VDR) to exert its biological effects and promote myogenic differentiation. Finally, 1,25(OH)2D enhances the expression of MyoD, which, in turn, inhibits myostatin production in a time-dependent manner (70). The active form of vitamin D, 1,25(OH)2D, is primarily synthesized in the kidneys via the mitochondrial enzyme 1α-hydroxylase, which is encoded by the Cyp27b1 gene. Vitamin D, widely recognized as a key regulator of bone metabolism, has long been a focal point in clinical interventions. However, its role in muscle tissue regulation has only recently garnered increased attention. Vitamin D replacement therapy has become well-established and may represent a promising therapeutic strategy for the treatment of OS.

### 4.4 Sex hormones

Disruption of sex hormone metabolism is considered one of the primary contributors to bone loss (71). Testosterone directly promotes osteoblast differentiation and indirectly facilitates it through its effects on various cytokines (72). Estrogen also exerts osteogenic effects, although to a lesser extent than testosterone. Numerous studies have demonstrated that the incidence of bone loss is significantly higher in elderly women compared to men. Postmenopausal women are particularly vulnerable to bone loss due to a decrease in the secretion of estrogen and progesterone from the ovaries. This results in estrogen deficiency, which accelerates bone turnover (73, 74). The increased activity of the basic multicellular unit (BMU) during bone remodeling leads to enhanced osteoblast apoptosis, shortened bone formation time, and reduced osteoclast apoptosis (75).

Additionally, sex hormones play a critical role in the metabolic processes of muscle tissue. In the cytoplasm, testosterone binds to androgen receptors and promotes protein synthesis through the mitogen-activated protein kinase (MAPK) pathway, thereby increasing muscle protein synthesis and muscle mass (76). Estradiol, on the other hand, facilitates muscle tissue repair through specific estrogen receptors (77). In chronic conditions such as cirrhosis, patients often exhibit reduced testosterone levels and an imbalance in the estrogen-to-androgen ratio. This imbalance leads to decreased osteoblast and osteocyte formation (78), reduced muscle protein turnover, and inhibition of myoblast differentiation into skeletal muscle cells (59), ultimately contributing to an imbalance in bone remodeling and a decline in muscle mass. The development of OS is closely associated with sex hormone deficiency, which is influenced by both aging and chronic diseases (60). It is important to note that while hormone replacement therapy is often associated with adverse effects, targeted therapies acting on specific receptors may offer a more promising therapeutic approach.

Current research on the pathogenesis of OS remains a subject of ongoing debate. Traditionally, osteoporosis and sarcopenia have been considered separate entities. However, recent studies have highlighted the significant interactions between bone and muscle tissues. A comprehensive understanding of the pathogenesis of both osteoporosis and sarcopenia is essential for advancing clinical knowledge. Furthermore, exploring the potential crosstalk between bone and muscle could offer valuable insights and novel therapeutic strategies for the prevention and treatment of OS.

### 4.5 Endocrine role of osteocytes and myocytes

#### 4.5.1 FGF-23

Fibroblast growth factor 23 (FGF-23) was the first bone-derived factor to be identified (79), primarily synthesized by osteoblasts. Elevated levels of FGF-23 were later discovered in the circulation of patients with rickets, leading to the recognition of its negative regulatory role in bone metabolism (80). The osteomalacia induced by FGF-23 is closely linked to the bone-kidney axis. FGF-23 primarily targets the proximal renal tubules, where it reduces the expression of co-transporters involved in phosphate absorption and reabsorption, thereby impairing bone deposition (81). Additionally, FGF-23 regulates vitamin D metabolism by inhibiting 1-α-hydroxylase, the enzyme responsible for converting 25-hydroxyvitamin D (25(OH)D) to its active form 1,25(OH)2D). This inhibition reduces the synthesis of 1,25(OH)2D, ultimately affecting bone mineralization (82). The Klotho protein acts as a downstream activator of the FGF-23 receptor pathway. Reduced expression of Klotho leads to elevated levels of FGF-23, which increases phosphate excretion in the proximal renal tubules (83). In CKD, Klotho levels gradually decline as the glomerular filtration rate decreases, often serving as one of the earliest indicators of disturbances in bone metabolism (84).

Moreover, Kido et al. found that FGF-23 is associated with muscle atrophy in CKD (85). The effects of FGF-23 on muscle may occur independently of s-Klotho and could directly interact with FGF receptors in skeletal muscle to exert inhibitory effects (86). Additionally, Chisato Sato et al. conducted an *in vitro* study to investigate the effects of FGF-23 on isolated human BMSCs. Their findings revealed that FGF-23 promotes the p53/p21/oxidative stress pathway, inducing premature senescence of human BMSCs in a Klotho-independent manner (87). In muscle tissue, BMSCs, which serve as the primary source of satellite cell differentiation, play a crucial role in maintaining skeletal muscle mass and repairing muscle fibers. In conclusion, osteocytes play a pivotal role in bone and muscle homeostasis. Unlike other members of the fibroblast growth factor (FGF) family, FGF-23 is almost exclusively produced in the skeletal system (79). BMSCs, which serve as precursors to osteoblasts, osteoclasts, and satellite cells, hold significant potential for repairing both bone and muscle damage. Further investigation into the effects of bone-derived, hormone-like factors such as FGF-23 on BMSCs may provide valuable insights and therapeutic strategies for addressing OS (88).

#### 4.5.2 Sclerostin

Sclerostin (28 kDa) is a small secretory glycoprotein synthesized by osteocytes and encoded by the SOST gene. It belongs to the Dan/Cerberus family of bone morphogenetic protein (BMP) antagonists (33). In a study by Graciolli FG et al. on CKD, serum sclerostin levels were found to increase as the disease progressed, leading to the inhibition of osteoblastogenesis by suppressing the WNT/β-catenin signaling pathway. Sclerostin also promotes bone resorption by inducing the synthesis of RANK-L, thereby enhancing osteoclastogenesis (89). Additionally, sclerostin interacts with BMP to inhibit BMP-induced Smad phosphorylation and disrupts the canonical Wnt pathway by competitively binding to the co-receptor of the Wnt pathway, LDL receptor-related protein 5/6 (LRP5/6) (90). Furthermore, studies have shown that hypoxia/HIF-1α, in collaboration with Osterix, inhibits the Wnt pathway, thereby suppressing osteoblast proliferation. Activation of HIF-1α induces sclerostin (Sost gene) expression, representing a novel mechanism by which HIF-1α impedes osteoblast Wnt signaling (91).

Recent studies have also emphasized the critical role of sclerostin in regulating muscle mass. Research by von Maltzahn et al. demonstrated that sclerostin inhibits muscle stem cell differentiation by suppressing the Wnt signaling pathway (92). Additionally, a study by Soohyun P. Kim et al. found that SOST gene knockout mice exhibited a significant increase in lean body mass compared to control groups (93). Moreover, it was observed that sclerostin suppresses the crosstalk between MLO-Y4 osteocytes and muscle cells (C2C12) mediated by WNT3a through the regulation of the Wnt/β-catenin pathway (94). These findings suggest that the effects of sclerostin on both bone and muscle are not merely independent signaling pathways. More importantly, osteocytes, as the primary source of sclerostin, may mediate the interactive signaling mechanisms between bone and muscle cells.

#### 4.5.3 Myostatin

Myostatin (Mstn), the first identified myokine, is primarily secreted by muscle fibers and functions as a negative regulator of skeletal homeostasis. *In vitro* studies have demonstrated that Mstn inhibits the expression of critical osteogenic transcription factors, such as Osterix and Runx2, thereby suppressing osteoblast differentiation (33). *In vivo*, Chen et al. observed that recombinant Mstn reduced the number of osteoblasts present on bone surfaces (95). Additionally, Qin et al. showed that Mstn upregulates the expression of Wnt pathway inhibitors, including SOST and Dickkopf Wnt signaling pathway inhibitor 1 (DKK1), which impedes osteocyte differentiation (96). Mstn also enhances the expression of RANKL, a pivotal gene involved in osteoclastogenesis, within osteocytes. However, in contrast to its effects on osteocytes, Mstn does not directly affect osteoclast activity. Instead, it promotes the expression of genes that mediate RANKL-induced osteoclastogenesis by facilitating SMAD2-dependent nuclear translocation of NFATc1, thus stimulating osteoclast proliferation independently of other signaling pathways (96).

As the name suggests, Mstn is inversely correlated with muscle mass. The regulation of muscle development by Mstn is complex, and although the precise mechanisms remain incompletely understood, it is generally accepted that Mstn mediates its effects through two primary signaling pathways: the MAPK pathway and the phosphoinositide 3-kinase (PI3K) pathway. Mstn downregulates the MEK/ERK1/2 MAPK pathway and/or the AKT/mTORC1 signaling cascade, leading to decreased expression of muscle-specific genes, such as Pax3, Myod1 (myogenic differentiation 1), and Myf5, thereby inhibiting myocyte differentiation (97). In addition, Mstn suppresses the persistent activation of eukaryotic translation initiation factor 4E (eIF4E) and eukaryotic translation initiation factor 4E-binding protein 1 (4E-BP1) within the PI3K/Akt/mTOR pathway, disrupting the balance between muscle protein synthesis and degradation, ultimately resulting in a reduction in myocyte cytoplasmic volume. Furthermore, Mstn promotes the expression of genes involved in the UPS, such as Murf-1, which accelerates muscle protein degradation (98).

Moreover, Mstn may act as a potential molecular mediator of bone-muscle loss in chronic diseases. For instance, uremic toxins, such as indoxyl sulfate, have been shown to elevate Mstn levels in CKD patients (99). Increased tumor necrosis factor-alpha (TNF-α) levels in chronic inflammatory diseases can also mediate the upregulation of Mstn via the NF-κB pathway, suppressing MyoD-induced Mstn expression (100). Additionally, IL-6 has been found to directly enhance Mstn expression in muscle fibers (30). It is noteworthy that Mstn also inhibits the differentiation of BMSCs into osteoblasts. Hamrick et al. demonstrated that Mstn may alter the mechanical responsiveness of BMSCs by suppressing the expression of osteogenic factors during mechanical loading (101). Additionally, Rebbapragada et al. showed that Mstn antagonizes bone morphogenetic protein (BMP) 7-induced adipogenesis, thereby inhibiting BMSC differentiation into adipocytes (102). BMPs play a pivotal role in regulating muscle growth and spatial patterning during embryonic development. The imbalance in BMSC differentiation is a key factor in the pathogenesis of bone density disorders, and genomic studies exploring adipogenic differentiation of BMSCs remain an active area of investigation. Recent research has identified DAAM2, TIMP2, and TMEM241 as potential therapeutic targets for bone degeneration and osteoporosis-related conditions (103). Furthermore, BMSCs, as precursors to satellite cells, are essential for muscle repair following injury. Although it remains uncertain whether the adipogenic imbalance of BMSCs directly influences satellite cell differentiation, this imbalance could represent a potential mechanism by which mesenchymal stem cells (MSCs) contribute to the treatment of bone-muscle loss syndromes.

#### 4.5.4 Irisin

Irisin is another myokine, but its effects differ significantly from those of Mstn. As a positive regulator of both the skeletal and muscular systems, irisin plays a crucial role in maintaining metabolic homeostasis (33). It can promote osteoblast proliferation and their differentiation into osteocytes via the MAPK signaling pathway (104). Furthermore, Storlino et al. demonstrated that intermittent administration of irisin downregulated the expression of sost and inhibited osteocyte apoptosis under conditions of oxidative stress and/or microgravity (105). Additionally, irisin stimulates the proliferation of osteoclast precursors and suppresses their differentiation through the p38, c-Jun N-terminal kinase (JNK) signaling pathways, as well as the RANKL-induced NF-κB pathway (106).

Irisin has also been shown to promote myogenesis through autocrine mechanisms. Specifically, it increases the mRNA levels of muscle growth-related genes, such as insulin-like growth factor 1 (IGF-1) and peroxisome proliferator-activated receptor gamma coactivator 1-alpha (Pgc1α4), primarily via the ERK signaling pathway. Moreover, irisin inhibits the expression of Mstn, thereby synergistically enhancing muscle protein synthesis (107).

**Figure 2:**
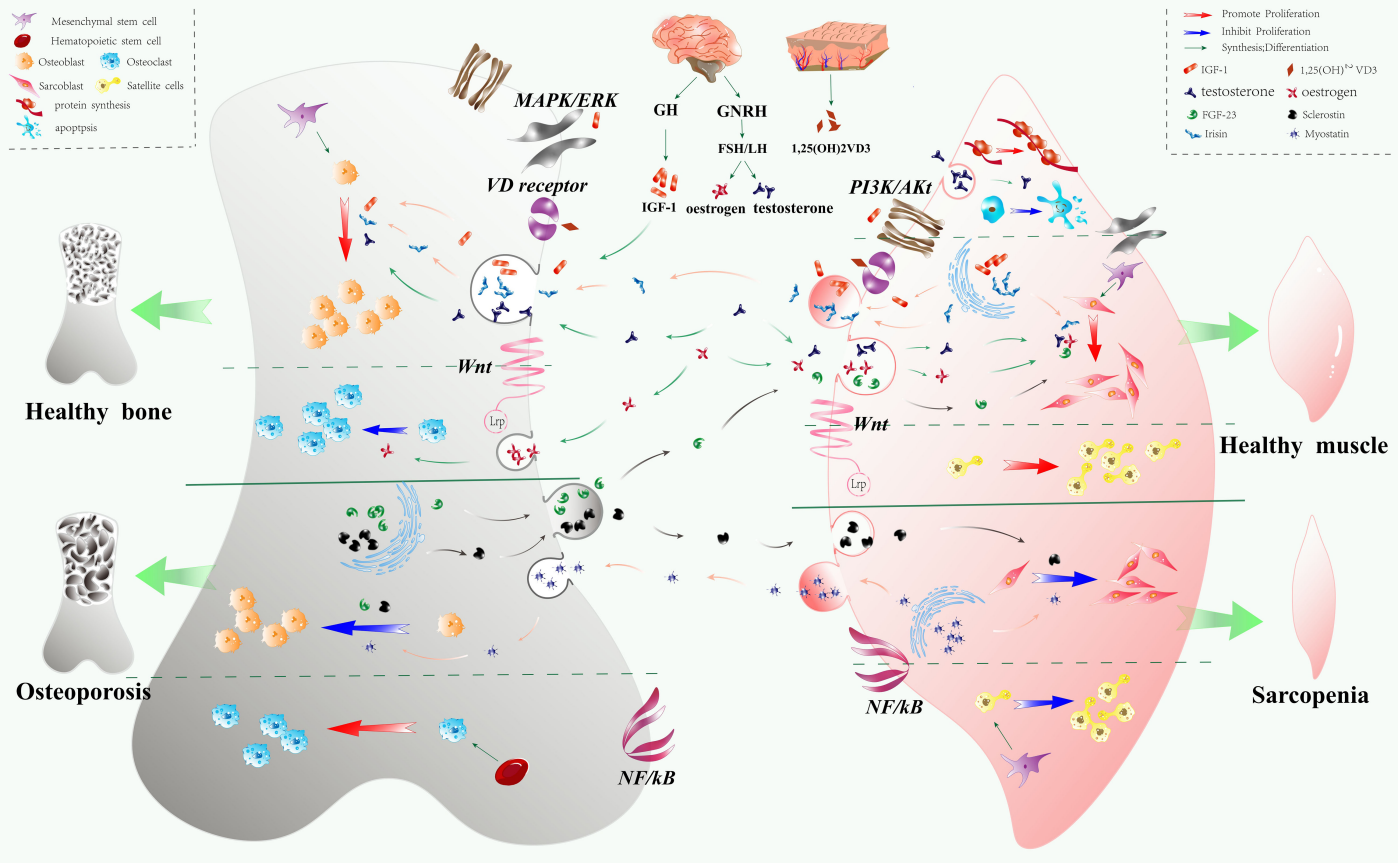
Interaction between the skeletal and muscular systems. The skeletal and muscular systems both derive from a shared mesenchymal stem cell lineage, with their proliferation and differentiation regulated by overlapping signaling pathways and cytokines. These systems function as “endocrine” organs, secreting factors that influence one another and contribute to the overall homeostasis of the musculoskeletal system. Positive interactions between bone and muscle are primarily mediated by pathways such as WNT, MAPK/ERK, PI3K/AKT, and the vitamin D receptor. In contrast, the NF-κB pathway serves as a negative regulator. Exogenous cytokines, including growth hormone/IGF-1, vitamin D, estrogen, and testosterone, promote the proliferation and differentiation of both bone and muscle cells, enhancing anabolic processes in these tissues. Bone-derived factors such as sclerostin and FGF-23, and muscle-derived factors like myostatin and irisin, regulate muscle catabolism, protein synthesis, and bone remodeling. Specifically, irisin promotes the proliferation of both bone and muscle cells, while sclerostin and myostatin inhibit cell proliferation. FGF-23 inhibits bone remodeling but positively influences muscle cell proliferation.

In the endocrine interaction pathways between osteocytes and muscle cells, it is evident that several shared pathways contribute to conditions resembling OS. Osteocytes play a pivotal role in the ‘bone-muscle crosstalk.’ Regardless of their origin—whether from bone or muscle—most factors activate downstream signaling pathways through osteocyte-mediated secretion or regulation, such as SOST and RANKL, which, in turn, influence the metabolic processes of both bone and muscle. A comprehensive understanding of the combined actions of these pathways could improve the management of conditions like OS. In conclusion, the development of OS is not solely driven by the mechanisms underlying osteopenia/osteoporosis or sarcopenia, but is more significantly influenced by the pathological and physiological interactions that perpetuate a vicious cycle between these two conditions (**Figure 2**).

**Figure 3:**
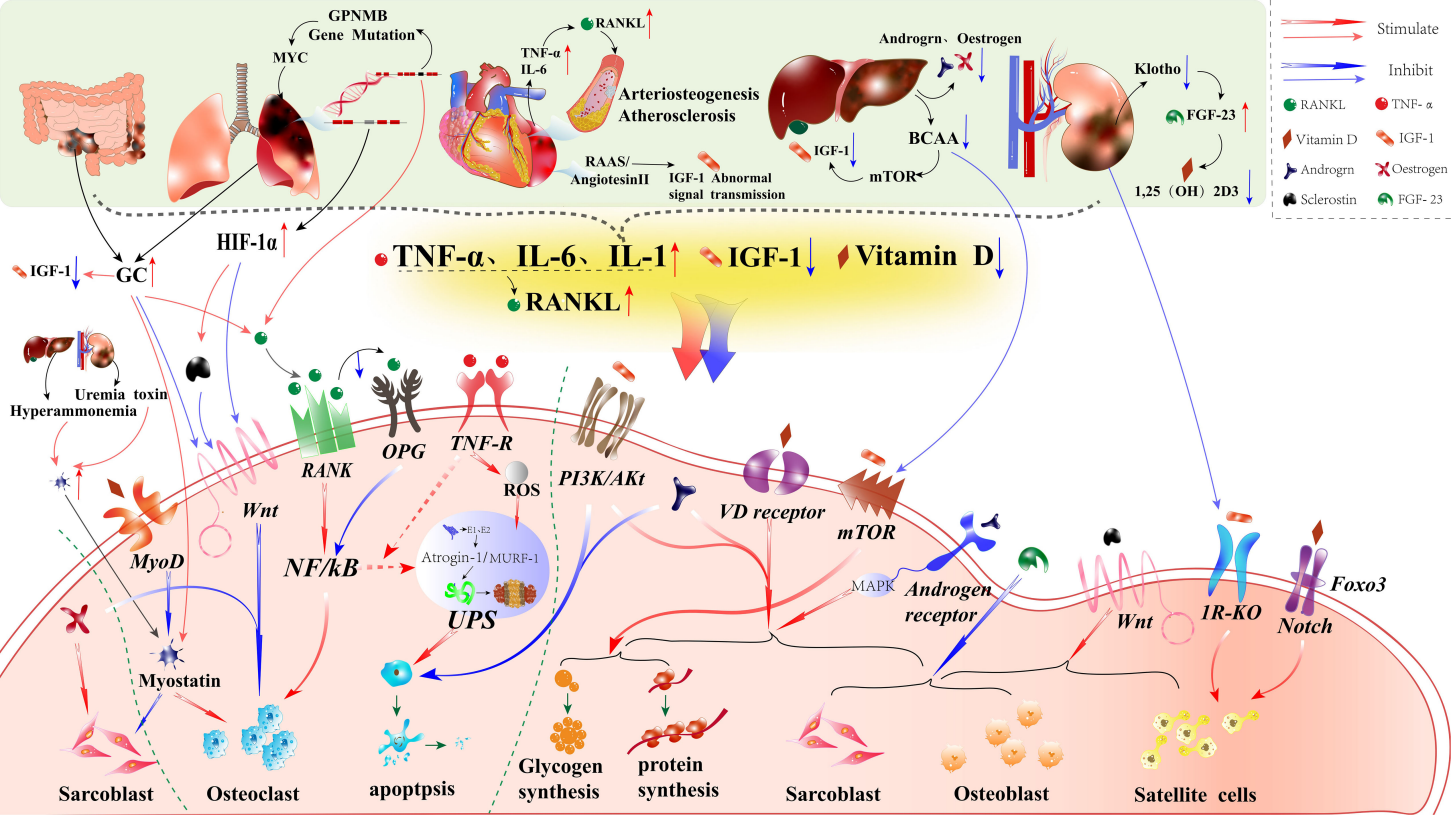
Pathophysiological processes of chronic diseases and their direct effects on shared molecular mechanisms in bone-muscle interactions. This figure illustrates the shared molecular mechanisms underlying bone-muscle interactions, with “→” indicating a positive effect and “→”indicating a negative effect. The green dashed line highlights the regulation of bone resorption and muscle catabolism, while other pathways emphasize the regulation of bone formation and muscle anabolic processes. The pathophysiological processes associated with chronic diseases and sarcopenia are depicted above, with “→” indicating a promoting effect and “→” indicating an inhibitory effect. Chronic diseases converge on these pathways through elevated inflammatory factors (e.g., TNF-α, IL-6, IL-1) and reduced levels of insulin-like growth factor 1 (IGF-1) and active vitamin D, negatively impacting the musculoskeletal system by promoting bone resorption and muscle catabolism. Specifically, inflammatory cytokines increase bone resorption and muscle breakdown, while decreased IGF-1 and vitamin D levels hinder bone formation and muscle anabolism, resulting in an imbalance in bone and muscle mass. Furthermore, pathological interactions vary across chronic diseases: in chronic obstructive pulmonary disease (COPD), mutations in the GPNMB gene and upregulation of HIF-1α expression; in inflammatory bowel disease (IBD) and COPD, glucocorticoid (GC) use; and in chronic kidney disease (CKD) and liver cirrhosis (LC), the accumulation of toxic substances (such as hyperammonemia and indoxyl sulfate) further promote bone resorption and muscle catabolism. Reduced branched-chain amino acids (BCAAs) in LC patients and decreased synthesis of active vitamin D (1,25(OH)_2_D_3_) in CKD patients inhibit anabolic processes, exacerbating musculoskeletal deterioration.

Indeed, OS does not solely occur in healthy adults but is a complex condition influenced by various factors, including inflammation, malnutrition, prolonged polypharmacy, frailty, functional impairment, and aging. While research on the mechanisms linking chronic diseases to OS remains limited, the interplay of common risk factors for both conditions provides indirect insights into the impact of chronic diseases on the development of OS (**Table 2**). In the long-term management of chronic diseases, MSK plays a crucial role, as the pathological mechanisms associated with these conditions contribute to the onset of OS. Therefore, OS should not be overlooked in patients with chronic diseases. It is a syndrome that coexists with and interacts with chronic diseases (**Figure 3**).

**Table 2:** The potential molecular mechanisms by which chronic diseases affect osteosarcopenia.

Study objective	Author/tear	Study type	Disease & potential mechanisms	Results	Cite
The Relationship Between Osteosarcopenia in Asymptomatic Individuals and Coronary Artery Calcification.	Chul-Hyun Park et al.;2022	Cross-Sectional Study	Coronary Artery Atherosclerosis & GH/IGF-1,RANK/RANKL‐OPG—NF-kB,NF-kB—UPS	The authors propose that the GH/IGF-1 axis plays a central role in the development of osteosarcopenia in coronary artery calcification. Another key mechanism is “inflammation,” where pro-inflammatory cytokines are highly associated with the development of osteosarcopenia through the ubiquitin-proteasome pathway, leading to accelerated bone resorption.	(38)
Factors Influencing Sarcopenia and Osteosarcopenia in Chronic Liver Disease and Their Molecular Pathogenesis.	Chisato Saeki et al.;2021	Review	Chronic Liver Disease & NF-kB,IGF-1,Vitamin D,Sclerostin,FGF-23,Myostatin and Irisin	The authors believe that pro-inflammatory cytokines (such as IL-1β, IL-6, and TNF-α), IGF-1, vitamin D, sclerostin, FGF-23, myostatin, and irisinin play crucial roles in the pathogenesis of osteosarcopenia in chronic liver disease.	(57)
Sarcopenia as a Comorbidity of Cardiovascular Disease.	Ken-ichiro Sasaki et al.;2022	Review	Heart Failure & RANK/RANKL/OPG—NF-kB,NF-kB—UPS,IGF-1 and BCAA	The authors suggest that inflammatory biomarkers in heart failure (HF), such as IL-6, TNF-α, and C-reactive protein, have a direct impact and influence the onset of osteosarcopenia by reducing IGF-1 levels and branched-chain amino acids. Oxidative stress activates the UPS system, promoting protein degradation. Additionally, low testosterone levels induced by HF also play a role.	(60)
An Overview of the Molecular Mechanisms Leading to Musculoskeletal Disorders in Chronic Liver Disease.	Young Joo Yang et al.;2021	Review	Chronic Liver Disease & RANK/RANKL‐OPG—NF-kB,IGF-1,BCAA and Testosterone	The authors highlight the role of the inflammation-mediated RANK/RANKL-OPG-NF-kB pathway in chronic liver disease, as well as the impact of hepatocyte damage leading to decreased levels of IGF-1, BCAAs, and testosterone in the development of osteosarcopenia.	(58)
Osteosarcopenia and Long-COVID: a dangerous combination	Umberto Tarantino et al.;2022	Review	COVID & RANK/RANKL‐OPG—NF-kB and NF-kB—UPS	The authors describe how chronic inflammation in long COVID mediates the RANK/RANKL-OPG-NF-kB pathway and the NF-kB-UPS system, influencing the development of osteosarcopenia.	(49)
Osteosarcopenia in Patients with Chronic Obstructive Pulmonary Disease (COPD).	Lorenzo Lippi et al.;2022	Review	COPD & RANKL—NF-kB,NF-kB—UPS and IGF-1	The authors state that long-term inflammation in COPD mediates the effects of NF-κB, RANKL (Receptor Activator of Nuclear Factor-kappa B Ligand), and M-CSF (Macrophage Colony-Stimulating Factor), while hypoxic damage activates the UPS system. Additionally, long-term hormone therapy leading to decreased IGF-1 levels plays a key role in the development of osteosarcopenia.	(39)
Osteosarcopenia in Chronic Pancreatitis.	I V Kozlova et al.;2021	Case-control study	Chronic Pancreatitis & RANK/RANKL‐OPG—NF-kB and NF-kB—UPS	The correlation between lumbar spine T-score and IL-6 (r = -0.29; p = 0.03) and IL-8 (r = -0.29; p = 0.04) was revealed. A correlation was also established between osteosarcopenia and cytokine concentrations in the colonic mucosa in chronic pancreatitis (IL-2: r = 0.44; p < 0.001; IL-6: r = 0.48; p < 0.001; IL-8: r = 0.42; p < 0.001).	(42)
Aromatase Inhibitor-Induced Osteoporosis and Osteopenia in Elderly Breast Cancer Patients.	Andrea Casabella et al.;2024	Case-control study	Breast Cancer & Estrogen and RANKL—NF-kB	The authors reveal that decreased estrogen secretion in elderly breast cancer patients leads to increased secretion of RANKL by osteoblasts, which in turn enhances osteoclast activity, resulting in accelerated bone remodeling.	(74)
Osteosarcopenia in Peritoneal Dialysis Patients.	Andrew Davenport et al.;2022	Retrospective Study	Chronic Kidney Disease & Vitamin D	The authors suggest that chronic kidney disease, leading to abnormalities in calcium-phosphate metabolism and vitamin D synthesis, may be a contributing factor to the development of osteosarcopenia.	(67)
Recent Advances in Vitamin D and Osteosarcopenia.	Olivier Bruyère et al.;2017	Review	Chronic Kidney Disease/Chronic Liver Disease & Vitamin D	The authors suggest that vitamin D metabolism is coordinated by the skin, liver, and kidneys. Chronic liver and kidney diseases may lead to vitamin D deficiency. Vitamin D deficiency enhances bone marrow adipogenesis and intramuscular fat deposition, thereby impairing skeletal function. In muscle tissue, vitamin D regulates calcium flux, mineral homeostasis, and signaling pathways that control protein synthesis and metabolism.	(68)
The Vicious Cycle of Osteosarcopenia in Inflammatory Bowel Disease.	Dorota Skrzypczak et al.;2021	Review	Inflammatory Bowel Disease & RANKL/NF-kB and Hormone(GC)	The authors state that long-term inflammation in patients with inflammatory bowel disease mediates the RANKL/NF-kB pathway and the use of hormones, which may contribute to the development of osteosarcopenia. They also emphasize the endocrine interaction between osteocytes and myocytes.	(4)

## 5 Management

### 5.1 Non-pharmacological management

Exercise is widely regarded as one of the most cost-effective and beneficial strategies for managing OS, offering significant advantages for patients with chronic diseases. Research indicates that physical activity can effectively prevent the onset of OS and alleviate its associated negative outcomes. For OS linked to chronic conditions such as cardiovascular diseases, diabetes, cancer, and respiratory disorders, exercise is commonly recommended as a therapeutic intervention. A 2023 statement from the American Heart Association underscores that resistance training not only enhances the quality and strength of the MSK but also provides essential physiological and clinical benefits for individuals with cardiovascular diseases and related risk factors (108). In clinical practice, all patients with chronic diseases diagnosed with OS should be prescribed resistance training (RT). RT may include various forms of exercise, such as free weights (e.g., dumbbells), bodyweight exercises (e.g., push-ups, squats), machine weights, or resistance bands. Each session should consist of 1 to 3 sets at a moderate intensity, with 8 to 12 repetitions per set performed to the point of fatigue, at least twice a week (108). Furthermore, RT should be sustained as a preventive measure for patients with chronic diseases.

Additionally, studies have emphasized the critical role of proper nutritional support in managing OS. Patients are advised to maintain a diverse and balanced diet that includes essential trace elements, vitamins, proteins, and dairy products. Increasing the intake of foods rich in BCAAs, such as meat (e.g., beef, salmon, shrimp), legumes, and nuts (e.g., peanuts, walnuts, almonds), is particularly beneficial (109). Simultaneously, the consumption of soft drinks and alcohol should be minimized. Furthermore, it is recommended that patients with OS consistently consume 1000 to 1500 mg of calcium daily, along with 400–800 IU of vitamin D or 260 µg of 25-hydroxyvitamin D supplements (e.g., calcitriol) every two weeks as part of their daily nutritional regimen (110). This combination provides pharmacological support to complement basic nutritional intake.

### 5.2 Pharmacological management

#### 5.2.1 Inflammation

In many chronic diseases, systemic inflammation persists throughout the disease course, playing a crucial role in long-term disease management (35). Celecoxib, a nonsteroidal anti-inflammatory drug (NSAID), has demonstrated effective anti-inflammatory properties in the MSK disorders and is commonly utilized in the clinical management of immune system diseases, osteoarthritis, cancer, cardiovascular diseases, and respiratory conditions. As a potential pharmacological approach for OS, celecoxib warrants further investigation. Additionally, the NF-κB pathway plays a pivotal role in the pathogenesis of OS. Furthermore, denosumab, a human monoclonal antibody that targets receptor activator of nuclear factor kappa-β ligand (RANKL), has been shown to effectively inhibit bone resorption and enhance bone mass. It is widely used in the treatment of osteoporosis (111). A study conducted over a three-year period, comparing women with OS, revealed that patients receiving denosumab treatment experienced significant improvements in both grip strength and lean body mass (112). These findings underscore the potential of denosumab as a promising therapeutic option for OS.

#### 5.2.2 Hormone therapy

Currently, hormone replacement therapies, including selective androgen receptor modulators (SARMs), selective estrogen receptor modulators (SERMs), growth hormone, and insulin-like growth factor 1 (IGF-1), are under extensive investigation for the treatment of OS. Mechanistically, these therapies hold promising potential in promoting both muscle and bone health. However, the clinical benefits have not always been consistently observed (58). Moreover, the adverse effects associated with hormone replacement therapies remain a concern, limiting their widespread use. Nevertheless, targeted therapy for patients with chronic diseases may offer more favorable responses. For instance, in patients with chronic liver disease, treatment with SERMs and branched-chain amino acid supplements may prove effective. In individuals with chronic kidney disease, a regimen including calcium, phosphate supplements, and vitamin D could provide potential benefits. Further clinical validation is required to substantiate these approaches.

#### 5.2.3 Endocrine targets in osteocytes and myocytes

Recent research on the management of OS has increasingly focused on the endocrine crosstalk between osteocytes and myocytes as a promising therapeutic target. Blosozumab, a human monoclonal antibody that targets sclerostin—an important regulator of bone metabolism—has shown considerable promise. A recent randomized, double-blind phase 2 clinical trial demonstrated that sclerostin inhibition with Blosozumab effectively treats osteoporosis in postmenopausal women, resulting in significant increases in bone mineral density (BMD) at the spine, femoral neck, and total hip (113). Additionally, ACE-031, a soluble form of activin receptor type IIB that functions as a Mstn inhibitor, can bind to and neutralize Mstn, thereby promoting muscle growth (114). A study demonstrated that ACE-031 was well tolerated, increased bone formation markers, and improved lean body mass in postmenopausal women (115). These findings suggest that both Blosozumab and ACE-031 may become viable treatment options for OS, particularly in patients with chronic diseases.

#### 5.2.4 Mesenchymal stem cell therapy

Mesenchymal stem cells (MSCs) are undifferentiated progenitor cells with self-renewal capacity and the ability to differentiate into multiple cell types, including adipocytes, osteoblasts, myocytes, and satellite cells (116). Due to their regenerative properties, MSCs are increasingly utilized in clinical settings, particularly for the management of chronic and long-term diseases (116). MSCs hold significant potential for the treatment of OS, with demonstrated abilities to promote osteogenesis and myogenesis, as well as repair damaged bone and muscle tissues. First, MSC transplantation is feasible, and over 1,000 MSC transplantation clinical trials have been registered, with human umbilical cord-derived MSCs being applied in multiple fields (117). Additionally, preclinical studies consistently demonstrate that MSCs improve bone density in animal models of osteoporosis, providing strong evidence for their efficacy (118). Furthermore, MSCs have been shown to promote muscle regeneration. Recently, induced pluripotent stem cells (iPSCs) have emerged as a potential source of stem cells. When used as autologous transplants, iPSCs can differentiate into muscle tissue and eliminate the risk of immune rejection (119). Furthermore, genetic manipulation of MSCs presents a promising strategy to enhance their therapeutic potential. Identifying specific genes that promote MSC differentiation into osteoblasts and satellite cells could enable more precise and efficient treatment of bone and muscle damage associated with OS. Additionally, metabolomics evaluations may provide valuable insights for diagnosing OS, particularly in individuals with genetic predispositions (120). However, despite these advancements, the clinical application of MSCs in the treatment of OS remains a significant challenge.

**Figure 4:**
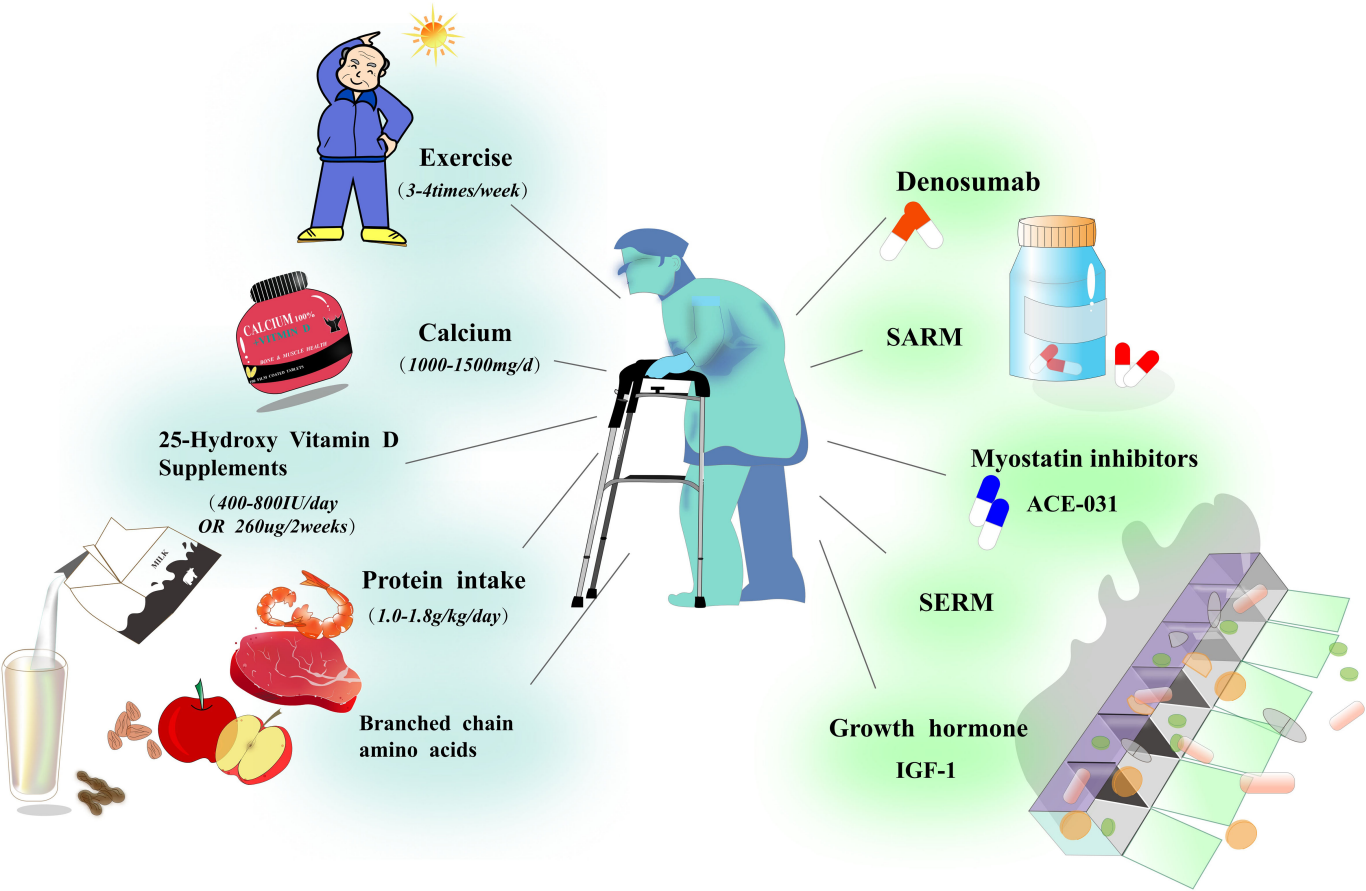
Prevention and treatment strategies for osteosarcopenia in patients with chronic diseases. This figure outlines key strategies for preventing and treating osteosarcopenia in patients with chronic diseases. Regular physical activity, including strength training and weight-bearing exercises, is essential for enhancing muscle mass and strength. Adequate nutrition, with sufficient daily intake of calcium, active vitamin D, dietary protein, and branched-chain amino acids (BCAAs), supports both muscle and bone health. Pharmacological treatments for osteosarcopenia include Denosumab (a monoclonal antibody targeting RANKL to prevent bone resorption), selective androgen receptor modulators (SARMs), selective estrogen receptor modulators (SERMs), growth hormone/IGF-1 therapy, myostatin inhibitors, and ACE-031 (an Activin receptor type IIB antagonist),Mesenchymal stem cell therapy.

Current research frequently addresses OS by treating osteoporosis and sarcopenia as distinct conditions. However, these approaches have demonstrated limited or no efficacy in slowing the progression of OS. Based on a comprehensive review of prior studies examining the “combinatory actions” between bone and muscle, we aim to identify promising strategies for the treatment of OS (**Figure 4**).

## 6 Conclusion

This review underscores the significant impact of OS on chronic diseases. As discussed, OS exacerbates adverse outcomes in chronic disease. With the rising prevalence of chronic conditions requiring long-term medical management, the strain on healthcare systems continues to intensify (7). However, despite its profound impact, OS has not received adequate attention in the long-term management of these diseases, and much of the research remains underdeveloped. Moreover, the consequences of OS on chronic diseases extend beyond the scope of this review. The musculoskeletal system, essential for energy production and movement, plays a pivotal role in maintaining overall health (10). Traditionally, chronic disease management has focused on bone loss and osteoporosis, but it is only in recent years that a more nuanced understanding of OS has emerged (1). Despite these advancements, current research continues to treat bone and muscle systems separately. In reality, the integrity of the MSK must be considered holistically. Therefore, it is crucial to urge clinicians to reconsider the critical role of OS in managing chronic diseases.

Additionally, this review delves into the potential mechanisms driving OS in chronic diseases. Moving away from the traditional approach of addressing bone and muscle systems independently, it highlights the concept of “bone-muscle crosstalk” mediated by osteocytes. The onset of OS is not merely the disruption of skeletal or muscular systems in isolation but rather the interaction between skeletal and muscular dysfunctions that, through “bone-muscle crosstalk,” exacerbates both bone loss and muscle atrophy (33) (36). By examining these mechanisms, we have integrated current prevention and treatment strategies, which may pave the way for advancements in the long-term management of chronic diseases.

Additionally, future recommendations are provided. Current research suggests that chronic diseases mediate the onset of OS through specific pathophysiological pathways. However, the precise role of OS in influencing the pathogenesis of chronic diseases remains a significant area of debate and an important research gap. Despite this, existing studies still indicate that, on a macro level, sarcopenia contributes to the increased prevalence or progression of chronic diseases. Future research, however, faces a significant challenge in exploring the underlying molecular mechanisms at the microscopic level.

## 7 Methodology

This systematic review was conducted in strict accordance with the Preferred Reporting Items for Systematic Reviews and Meta-Analyses (PRISMA) (121) guidelines.

A comprehensive literature search was performed in two electronic databases—PubMed and Google Scholar—covering studies published from January 2019 through January 2025. Notably, several of the included review articles incorporated data from high−quality studies conducted prior to 2010.The search strategy combined Medical Subject Headings (MeSH) terms with free−text keywords, including OS, osteoporosis, sarcopenia, bone mass loss, muscle mass loss, epidemiology, molecular mechanisms, management, chronic disease, and chronic inflammatory disease. Boolean operators (AND, OR) were applied to refine and expand the search results.

**Figure 5:**
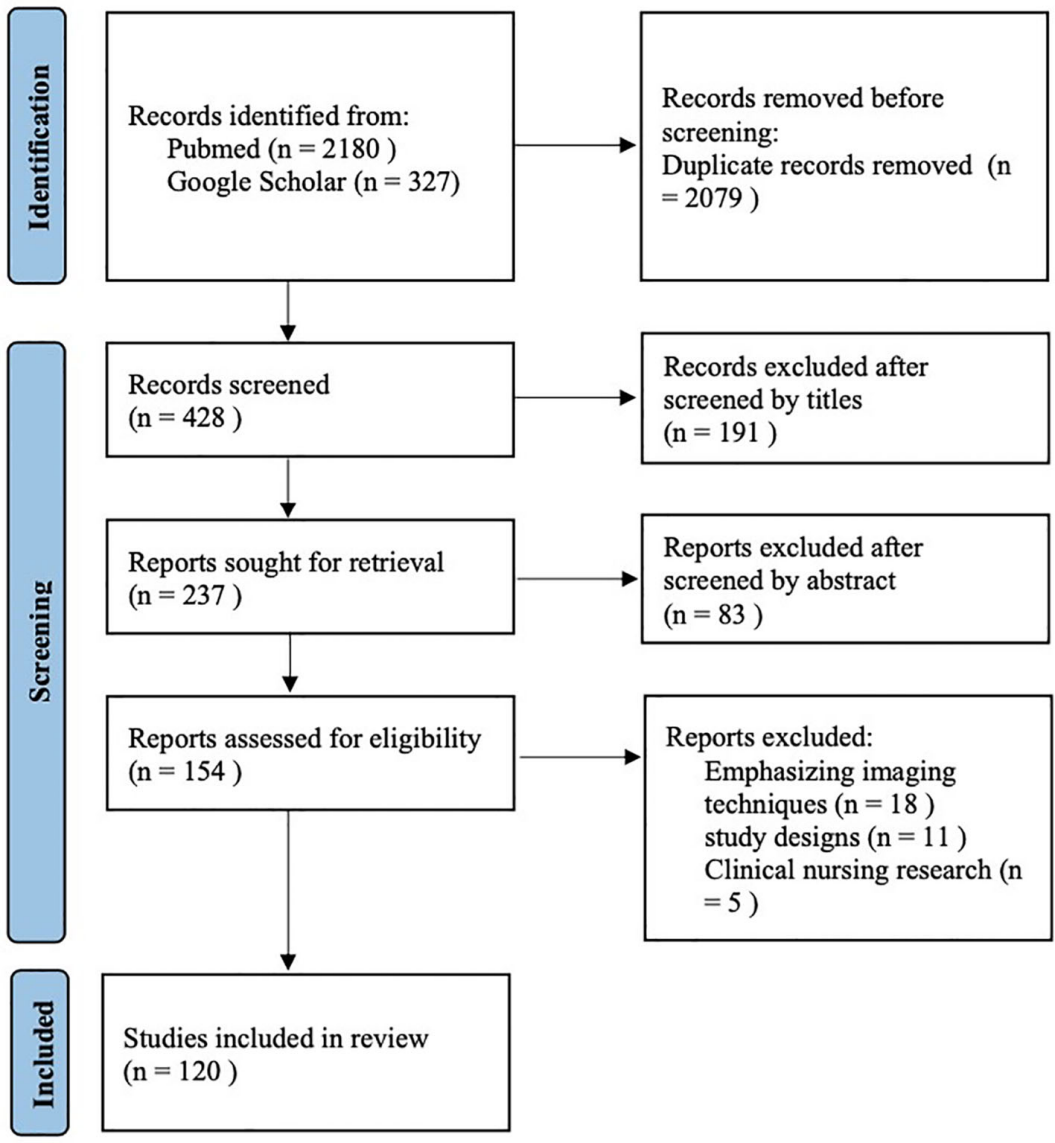
Flow diagram of the study selection process.

In addition, the reference lists of all retrieved articles were manually screened to identify any additional eligible studies. Two authors (YT−Y and HF−Z) independently screened titles and abstracts according to predefined inclusion criteria. Full texts were then reviewed to remove duplicates and exclude studies that did not meet the eligibility criteria. Any disagreements were resolved through discussion with a third reviewer (WZ−W). Eligible study designs included observational studies (cohort, case–control, and cross−sectional), systematic reviews, meta−analyses, and randomized double−blind controlled trials (RCTs). Animal studies and experimental model studies were excluded. Studies deemed irrelevant to the research focus of this review were also excluded. Additionally, case reports, editorials, letters to the editor, and conference proceedings were excluded. Ultimately, a total of 120 references were included in this review (**Figure 5**).
